# Onset of juvenile dermatomyositis following varicella infection in a 12-month-old child: a case report

**DOI:** 10.1186/1752-1947-8-54

**Published:** 2014-02-15

**Authors:** Nadine McCrea, Afraa Al-Sabbagh, Soliman Ahmed, David Walker, Satyapal Rangaraj

**Affiliations:** 1 Paediatric Neurology, Addenbrooke’s Hospital, Cambridge CB2 0QQ, UK; 2 Paediatrics, Peterborough City Hospital, Edith Cavell Campus, Bretton Gate, Peterborough PE3 9GZ, UK; 3 Radiology, Peterborough City Hospital, Peterborough, UK; 4 Paediatric Oncology, Faculty of Medicine and Health Sciences, Nottingham Children’s Hospital, University of Nottingham, Nottingham NG7 2UH, UK; 5 Paediatric Rheumatology, Nottingham Children’s Hospital, Nottingham University Hospital NHS Trust, Nottingham NG7 2UH, UK

**Keywords:** Child, Dermatomyositis, Human herpesvirus 3, Infant, Precipitating factors, Preschool child

## Abstract

**Introduction:**

Infections can act as a trigger for juvenile dermatomyositis, with a predominance of respiratory tract infections reported previously. We present the first case of juvenile dermatomyositis following varicella infection to be described in the literature.

**Case presentation:**

A 15-month-old Caucasian girl was diagnosed with juvenile dermatomyositis 3 months after a varicella infection. The diagnosis was challenging due to her young age, but was supported by magnetic resonance imaging, and confirmed following a later appearance of the characteristic skin rash.

**Conclusion:**

Varicella infection may be a trigger for juvenile dermatomyositis. Further understanding of disease triggers is required.

## Introduction

Juvenile dermatomyositis (JDM) is a rare autoimmune inflammatory myositis. Micro-angiopathic changes affect muscle, skin and small nerves, amongst other tissues. The predominant clinical features are proximal muscle weakness, a characteristic skin rash, and constitutional symptoms. Genetic factors and environmental triggers are likely to play important roles in aetiology, although the exact mechanisms are not fully understood. There is a higher incidence of retrospective parent-reported infective illnesses (mainly upper respiratory and gastrointestinal tract) in the months prior to the onset of JDM [1], but case–control studies often do not reveal higher rates of positive blood serology [2,3]. One case–control study did reveal a significantly higher rate of group A beta-haemolytic streptococcal (GABHS) disease in children with JDM; it is thought that molecular mimicry between the GABHS antigens and the myosin heavy chain may be the mechanism for an increased immune response that triggers disease [3]. There are no previously reported cases of JDM following varicella infection in the literature.

## Case presentation

**Figure 1:**
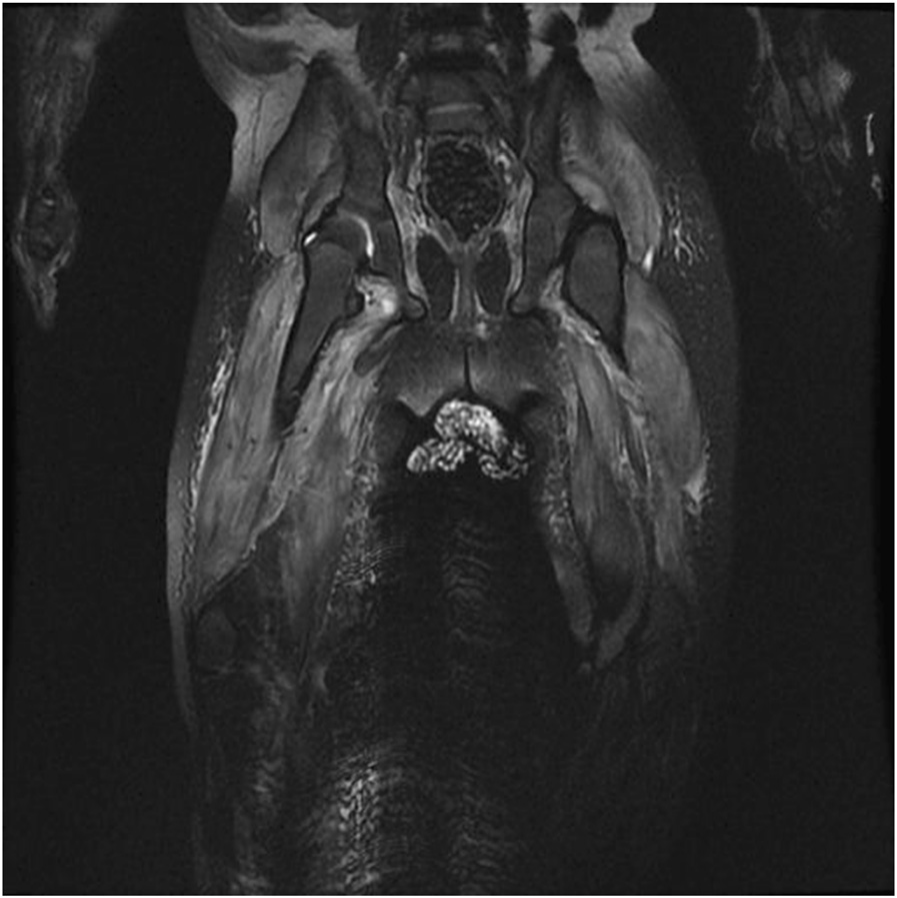
Coronal T2 magnetic resonance imaging sequence of upper legs, showing muscle and subcutaneous oedema.

A 15-month-old Caucasian girl presented with a 3-month history of regression in motor milestones. She had been previously fit and well, and had been cruising around furniture at 11 months of age. At 12 months she had a bout of chickenpox, and subsequently became lethargic and weak. She gradually lost gross motor skills, becoming unable to walk, stand, or even sit unsupported. Her mother described a persistent low grade fever, and noticed that her eyes appeared puffy. On examination there was marked proximal muscle weakness and hypotonia. She was not able to sit without support, could not roll over, and there was marked head lag when she was pulled to sit from the supine position. She exhibited little spontaneous movement, but when propped up in a sitting position she could play with toys placed in front of her. Her deep tendon reflexes were difficult to elicit. She had periorbital oedema and erythema, but there were no other cutaneous signs. She was miserable. The diagnosis of JDM was suspected, even though her age was young and initial investigations were inconclusive: laboratory investigations revealed only mildly elevated muscle enzymes (creatine phosphokinase 303U/L, alanine transaminase 104U/L, aspartate transaminase 93U/L, lactate dehydrogenase 461U/L), normal inflammatory markers, and normal/negative complement C3 and C4, rheumatoid factor, anti-neutrophil cytoplasmic antibody and anti-nuclear antibody nerve conduction studies and electromyography were within normal limits; varicella zoster serology was positive. A magnetic resonance imaging (MRI) scan of her thigh muscles was performed, along with imaging of her brain and spine, and further neurometabolic investigations were planned. However, these were not required because the MRI showed marked muscle and subcutaneous oedema, supporting the diagnosis of myositis (Figure 1). Treatment with methylprednisolone was initiated, followed with long-term methotrexate and prednisolone. Subsequently the diagnosis of JDM was confirmed as her heliotrope rash became more pronounced, and she developed Gottron’s papules and nail fold telangiectasia. She made a gradual improvement, and at 9 months post-diagnosis is able to crawl, pull herself to stand and walk independently.

## Discussion

Varicella infection has been implicated as a trigger in other immune-mediated conditions, such as type 1 diabetes mellitus. We postulate this infection could have acted as a precipitating factor for JDM in this child.

This is one of the youngest children to have been diagnosed with JDM in the UK [4]. The mean age of onset of JDM in the UK is 6.3 years, however, in around one-third of cases the disease begins at the age of 4 years or younger [4]. Although JDM is rare in very young children, onset of symptoms prior to 12 months of age has been described [4,5]. Negative initial investigations should not cause JDM to be ruled out as a diagnosis, as muscle enzymes and electromyography can be normal. Indeed, in a review of 175 children with JDM no single test was consistently abnormal [6]. MRI is now a key investigation in this condition, and often tends to replace muscle biopsy [7].

## Conclusions

Varicella infection may act as a precipitating factor in JDM. In JDM, as in other immune-mediated conditions, further understanding of disease triggers and how the subsequent immune dysregulation can be targeted is required. A high index of suspicion is needed in order to prevent delays in the diagnosis of JDM because it can present in very young children and infants, cutaneous features may be nonspecific initially, and no single test is pathognomonic.

## Consent

Written informed consent was obtained from the patients for publication of this manuscript. A copy of the written consent is available for review by the Editor-in-Chief of this journal.

## Abbreviations

GABHS: Group A beta-haemolytic streptococcus; JDM: Juvenile dermatomyositis; MRI: Magnetic resonance imaging.

## Competing interests

The authors declare that they have no competing interests.

## Authors’ contributions

NM performed the literature review, wrote the first draft of the manuscript, and approved the final version. AAS, SA, DW and SR all revised the manuscript and approved the final version.
